# Parallel and costly changes to cellular immunity underlie the evolution of parasitoid resistance in three *Drosophila* species

**DOI:** 10.1371/journal.ppat.1006683

**Published:** 2017-10-19

**Authors:** John E. McGonigle, Alexandre B. Leitão, Sarah Ommeslag, Sophie Smith, Jonathan P. Day, Francis M. Jiggins

**Affiliations:** Department of Genetics, University of Cambridge, Cambridge, United Kingdom; Monash University, AUSTRALIA

## Abstract

A priority for biomedical research is to understand the causes of variation in susceptibility to infection. To investigate genetic variation in a model system, we used flies collected from single populations of three different species of *Drosophila* and artificially selected them for resistance to the parasitoid wasp *Leptopilina boulardi*, and found that survival rates increased 3 to 30 fold within 6 generations. Resistance in all three species involves a large increase in the number of the circulating hemocytes that kill parasitoids. However, the different species achieve this in different ways, with *D*. *melanogaster* moving sessile hemocytes into circulation while the other species simply produce more cells. Therefore, the convergent evolution of the immune phenotype has different developmental bases. These changes are costly, as resistant populations of all three species had greatly reduced larval survival. In all three species resistance is only costly when food is in short supply, and resistance was rapidly lost from *D*. *melanogaster* populations when food is restricted. Furthermore, evolving resistance to *L*. *boulardi* resulted in cross-resistance against other parasitoids. Therefore, whether a population evolves resistance will depend on ecological conditions including food availability and the presence of different parasite species.

## Author summary

We have found that three species of fruit fly evolve resistance to parasitic wasps (parasitoids) by increasing investment in their immune defences but they achieve this in different ways. Resistance always involved increases in the number of the circulating hemocytes, which are the blood cells that kill parasitoids. However, one species moved sessile hemocytes into circulation while the other species simply produce more cells. These changes are extremely costly, which explains why these species are susceptible to parasitism in nature. Whether a population evolves resistance depends on ecological conditions, as resistance is only costly when food is in short supply, and evolving resistance to one parasite can have the added benefit of providing resistance to other parasites.

## Introduction

Considerable genetic variation in susceptibility to infection exists both within and between populations [1]. This variation determines the burden of disease within populations, and represents the raw material from which populations can evolve resistance in nature and during the selective breeding of plants and animals. Insects are no exception to this pattern, and it is common to find highly resistant and susceptible genotypes within the same population [2,3]. Here, resistance can increase the survival of beneficial species like bees and make disease vectors less likely to transmit infection, or cause biological control programs to fail if insect pests evolve resistance. Aside from its economic and health impact, this variation provides a powerful tool for evolutionary biologists to understand the coevolution of hosts and parasites, and immunologists to understand the functioning of immune systems.

A priority for infectious disease research is therefore to understand why variation in disease susceptibility is maintained in populations and what the physiological basis of this variation is. In cases where parasites act as a strong selective pressure on populations, natural selection is expected to eliminate susceptible alleles, reducing genetic variation for resistance [4–7]. However, genetic variation can be maintained if there is a cost to possessing and maintaining the machinery of resistance [8]. There are two types of resistance cost. Inducible costs are caused when a successful immune response is mounted, and therefore only affect infected individuals. Constitutive costs are associated with possessing and maintaining resistance machinery, and are therefore borne even by uninfected individuals [4].

Constitutive costs associated with resistance have been identified in many taxa, including plants [9], insects [3,8,10–12] and mammals [13]. In times or places where the parasite pressure is low, constitutive costs can result in susceptible alleles being favoured [4,14]. This balance of costs and benefits can maintain variation in resistance within and between populations, via extrinsic ecological factors that cause variation in parasitism rates. Furthermore, because the prevalence of infection may decline in resistant populations, constitutive costs can also result in negative frequency-dependent selection (NFDS) where the fitness of an allele declines as its frequency increases. This can maintain genetic variation in populations and potentially cause resistance alleles to rise and fall in frequency as they ‘chase’ changes in the parasite population [6,9,15].

What physiological processes underlie constitutive costs to resistance? The production of resistance machinery may require the investment of limited resources [16,17]. Therefore, in the absence of a parasite, resistant individuals who pay upkeep on increased arsenals are at a selective disadvantage [18]. Moreover, immune effectors can cause collateral damage to self [19,20]. Here, resistant individuals, who are likely to possess larger immune arsenals, may be more likely to suffer auto-immune damage as they have more weaponry capable of misfiring [21,22]. Additionally, there is a myriad of other potential pleiotropic effects, where a genetic change that increases resistance has deleterious effects on some other physiological or developmental process [23–25]. If the selective pressure is sustained for long enough the cost to resistance might be lost [25]. For instance, in a number of cases insects that evolved costly insecticide resistance later lost these costs when either resistance alleles which were less costly spread through populations, or modifiers that reduced the cost spread [25,26].

One example where resistance has been associated with constitutive costs comes from *Drosophila melanogaster* and its parasitoid wasps [3,27]. Parasitoids are insects that lay their eggs inside or on the body of other arthropods. If the host cannot mount a successful immune response, the parasitoid larva feeds on it and ultimately kills it [28,29]. Parasitoid wasps are of great ecological importance in *Drosophila* [30–37], with mean parasitoid infection rates reaching 75% in some localities [38]. One parasitoid species from the Braconidae family—*Asobara tabida*—and two from the Figitidae family—*Leptopilina boulardi* and *L*. *heterotoma*—are held to be the most ecologically important larval parasitoids of *Drosophila melanogaster* [33], and exert a tremendous selective pressure on *Drosophila* larvae to avoid and combat parasitism [39]. In the *D*. *melanogaster* sub-group there is considerable variation in parasitoid resistance within and between populations [40] and species [41]. This variation has been used to select populations for higher resistance to *A*. *tabida* and *L*. *boulardi* [3,27]. In these populations resistance increased rapidly, but this gain came with a trade-off of reduced larval competitive ability when parasitoids are absent [3,27]. Selected populations also had reduced feeding rates relative to controls, suggesting an association between the ability to obtain resources and the cost of resistance [12].

*Drosophila*’s immune defence against parasites consists of a specialised cellular response called encapsulation. This response is dependent on the three mature hemocyte (blood cell) types found in *Drosophila*: plasmatocytes, crystal cells and lamellocytes [22]. Lamellocytes are rarely found in healthy larvae but are induced in high numbers during parasitoid infection [42,43]. In homeostasis, the majority of plasmatocytes and crystal cells are adherent to the larval epidermis in sessile patches but they can also be found in circulation in the hemolymph and in a specialized hematopoietic organ called the lymph gland [44]. During encapsulation plasmatocytes detect and form a first layer of cells around the parasitoid egg that is then fully enclosed by lamellocytes [45]. This capsule is then melanised by a phenol-oxidase protein cascade, dependent on crystal cells and lamellocytes, killing the unhatched wasp larva [46]. If the encapsulation response is not quick enough or if the response is disrupted or overwhelmed, the developing wasp larva kills the host [47,48].

A positive correlation between circulating hemocyte numbers and resistance to *A*. *tabida* between different species suggests that hemocyte concentration is a crucial factor for resistance [41]. Moreover, artificial selection for *A*. *tabida* resistance results in populations having twice the number of hemocytes in circulation [49]. However, resistance strategies to different parasitoids appear to vary. *D*. *melanogaster* populations selected for resistance to *L*. *boulardi* showed increased ability to encapsulate both *L*. *boulardi* and *A*. *tabida* [50]. In contrast, populations selected for resistance to *A*. *tabida* showed no significant increase in their capability to encapsulate *L*. *boulardi*. A possible explanation for this is that *L*. *boulardi* injects fly larvae with venoms that sabotage the immune response, and thus requires resistance to these venoms. As such, there may be both specific and common components of parasitoid resistance [50].

We have selected three species from the *Drosophila melanogaster* sub-group (*D*. *melanogaster*, *D*. *simulans*, *D*. *mauritiana*) for resistance to the parasitoid wasp *L*. *boulardi*. Each species was collected from a single geographical location. In all three species there was a rapid increase in the rate at which parasitoids were encapsulated in response to selection that was accompanied by increases in the number of circulating hemocytes and cross-resistance to *A*. *tabida*. This result suggests that higher circulating hemocyte numbers is the common component for parasitoid resistance. However, the physiological basis of this increase was different—in *D*. *melanogaster* sessile hemocytes had moved into circulation, while in the other species the total number of hemocytes was increased. In all three species resistance was extremely costly, with resistant populations suffering a considerable drop in their competitive ability. We conclude that across multiple species evolving resistance to parasitoid wasps requires costly investment in cellular immunity, and this is likely the reason why in nature many species have remained susceptible and suffer high levels of mortality due to parasitoid attack.

## Results

### Artificial selection results in rapid increases in the resistance of three Drosophila species to parasitoid wasps

**Fig 1:**
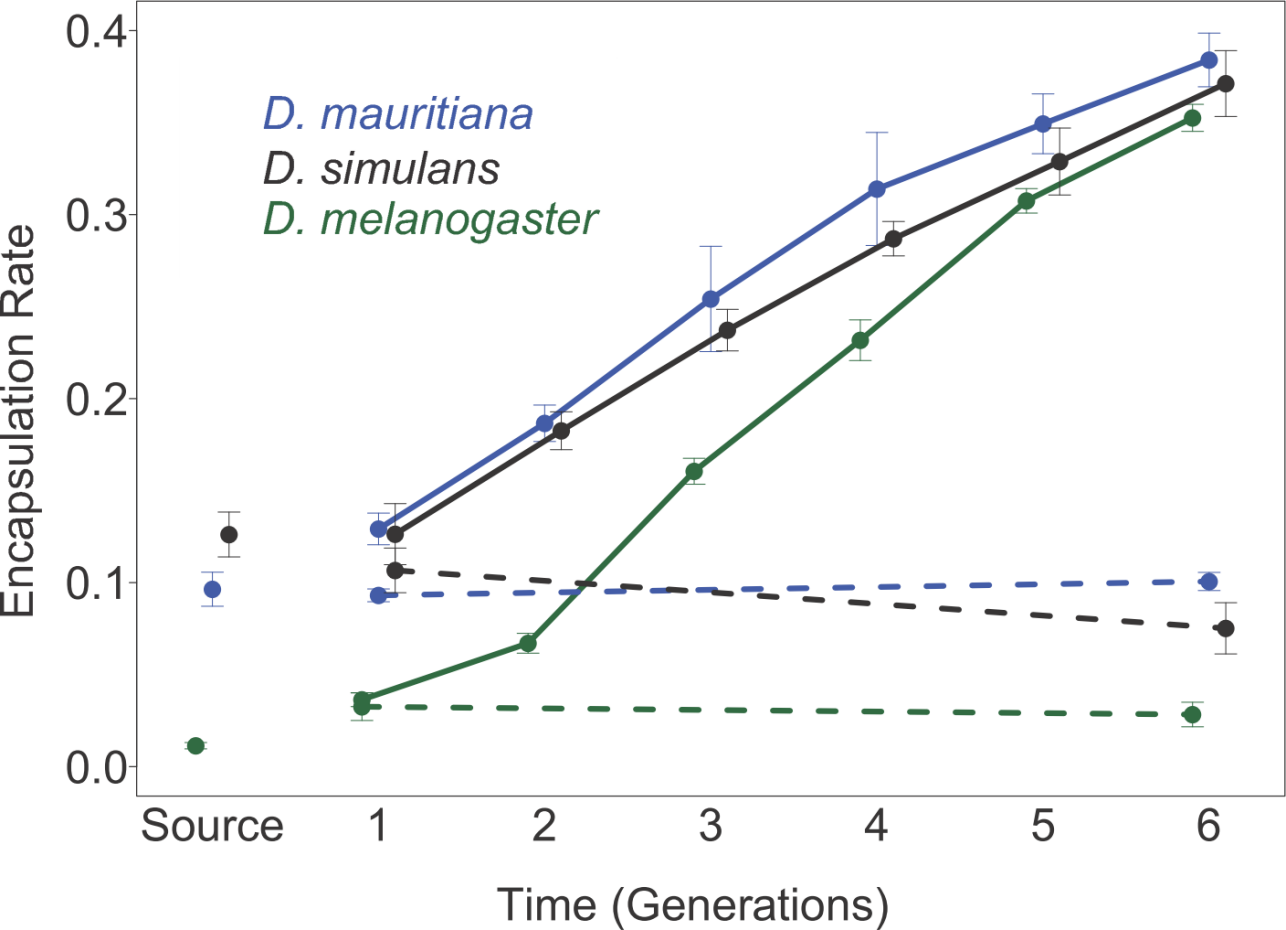
The proportion of parasitized larvae encapsulating the parasitoid wasp *L*. *boulardi* over six generations of selection. Three species of the *D*. *melanogaster* sub-group were selected for resistance over six generations and the encapsulation rate measured. Solid lines represent selected populations, while dotted lines represent control populations. Points are means of 6 replicate populations of *D*. *melanogaster* and *D*. *simulans*, and 3 replicate populations of *D*. *mauritiana*. Approximately 20 vials of flies were assayed per replicate population. Encapsulation rates were estimated using Eq 2 and the bars are equal to ±1 standard error (SE). The SE was calculated from the between replicate population variance for each species. This was impossible for the source population and so instead the mean’s bootstrap standard error is shown. ‘Source’ is the encapsulation rate of the population from which each set of the selection populations was founded, while generation one is the encapsulation rate following one generation of selection. Note that the assays on different species were carried out at different times; as such, precise comparisons between species should be interpreted with care.

To understand how different species evolve resistance to parasitism, we artificially selected three species of *Drosophila* for resistance to the parasitoid wasp *L*. *boulardi*. We only sampled a single population of each species—the population of *D*. *melanogaster* was from England, *D*. *simulans* from North America and *D*. *mauritiana* from Mauritius. The level of resistance initially varied greatly between species—only 1.1% of parasitised *D*. *melanogaster* larvae successfully encapsulated wasp eggs, compared to over 10% of *D*. *mauritiana* and *D*. *simulans* (Fig 1; Table A in S1 Supporting Information). Over six generations we exposed populations of these flies to parasitoids and retained only flies that mounted a successful immune response. As a control we maintained similar-sized populations of unparasitised flies. In total we parasitized approximately 3.5 million larvae in these experiments, and created three selected and three control populations for *D*. *mauritiana*, and six selected and six control populations for *D*. *melanogaster* and *D*. *simulans*.

In all three species, artificial selection resulted in a substantial increase in the ability of flies to encapsulate invading *L*. *boulardi* eggs (Fig 1; Table A in S1 Supporting Information). Despite the initial susceptibilities of the three species varying widely (Fig 1), following six generations of selection all three species encapsulated between 35–38% of parasitoids (Fig 1; Table A in S1 Supporting Information). Thus, encapsulation rates increased by approximately three fold in *D*. *simulans* and *D*. *mauritiana*, and approximately thirty fold in *D*. *melanogaster* (Mean encapsulation rates at generation 1 vs. 6. *D*. *melanogaster*: *t* = 32.7, *d*.*f*. = 10, *p* = <0.001; *D*. *simulans*: *t* = 12.9, *d*.*f*. = 10, *p* = <0.001; *D*. *mauritiana*: *t* = 18.5, *d*.*f*. = 4, *p* = <0.001). In contrast, control populations displayed no significant change in encapsulation rate over the six generations (Mean encapsulation rates at generation 1 vs. 6: *D*. *melanogaster*: *t* = -0.4, *d*.*f*. = 10, *p* = 0.689; *D*. *simulans*: *t* = -1.7, *d*.*f*. = 10, *p* = 0.126; *D*. *mauritiana*: *t* = 18.5, *d*.*f*. = 1.3, *p* = 0.272).

### Parasitoid resistance is associated with an increase in circulating hemocytes numbers

**Fig 2:**
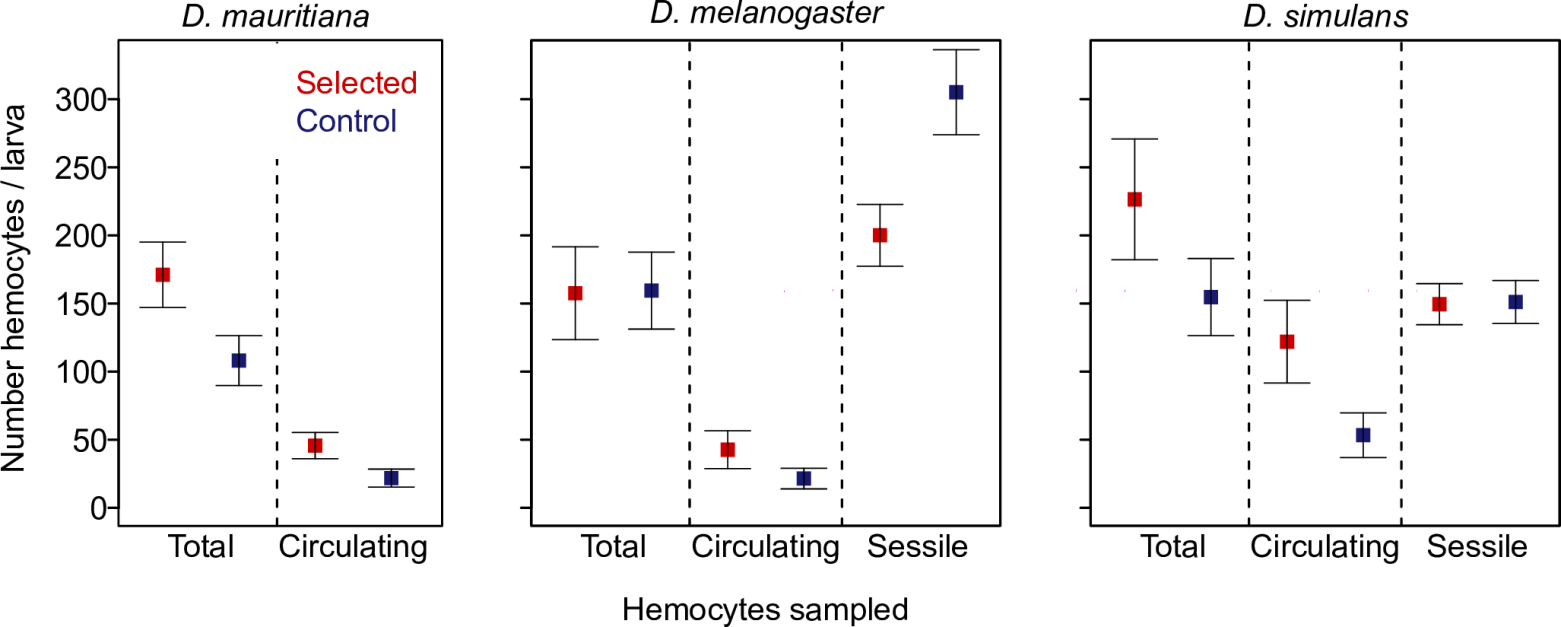
Mean number of hemocytes in populations of three *Drosophila* species that had been selected for parasitoid resistance and controls. The number of circulating hemocytes, the total number of hemocytes, and the number of sessile haemocytes under the dorsal cuticle. Hemocytes were counted in single 2^nd^ instar larvae. Estimates of total hemocyte numbers were achieved by agitating larvae to disrupt sessile clusters of hemocytes into circulation prior to bleeding. The sessile hemocytes were counted *in vivo* by injecting *E*.*coli* BioParticles. These are fluoresce when phagocytosed by hemocytes, allowing the cells to be seen under the cuticle of live larvae. This was done in a separate experiment and at a different generation post-selection than the other data. Points are the mean number of hemocytes and bars are equal to 1 SE. The mean number of larvae contributing to each point on the plot is 145.

Populations of *D*. *melanogaster*, *D*. *mauritiana* and *D*. *simulans* that were selected for parasitoid resistance were all found to possess more circulating hemocytes than control populations (Fig 2; *D*. *melanogaster*: *t* = 4.1, *d*.*f*. = 1, 190, *p* = 0.001; *D*. *simulans*: *t* = 3.7, *d*.*f*. = 1, 132, *p* = <0.001; *D*. *mauritiana*: *t* = 3.7, *d*.*f*. = 1, 88, *p* = <0.001). There was a 113% increase in circulating hemocyte number in *D*. *simulans*, an 84% increase in *D*. *melanogaster* and an 88% increase in *D*. *mauritiana*. This indicates a strong link between circulating hemocyte number and ability to resist invasion by a parasitoid wasp egg in all three species.

### Different physiological mechanisms underlie the increase in circulating hemocytes in different species

In *Drosophila* a large proportion of hemocytes are found in sessile clusters [52], so the increase in the number of circulating hemocytes seen in selected populations could either result from an increase in the total number of hemocytes or from hemocytes moving from sessile clusters into circulation. To distinguish between these hypotheses, we both estimated the total number of hemocytes per larva and counted sessile hemocytes under the cuticle.

To count the total number of hemocytes in each larva (sessile + circulating), we physically disrupted the sessile hemocytes before bleeding larvae. Populations of *D*. *mauritiana* and *D*. *simulans* selected for parasitoid resistance both had approximately 40% more total hemocytes than control populations (*D*. *simulans*: *t* = 3.7, *d*.*f*. = 1, 94, *p* = <0.001; *D*. *mauritiana*: *t* = 3.5, *d*.*f*. = 1, 86, *p* = <0.001). This increase indicates that resistance in these species may be achieved by increasing the pool of immune cells on which the body can draw. In contrast, larvae from the selected and control populations of *D*. *melanogaster* had very similar total numbers of hemocytes (*D*. *melanogaster*: *t* = 0.3, *d*.*f*. = 1, 254, *p* = 0.738). This is confirmed by examining the interaction between whether a population was selected or not and the number of total versus sessile hemocytes. In *D*. *melanogaster* this interaction was significant, indicating that the proportion of hemocytes in circulation had increased in the resistant populations (Fig 2; *D*. *melanogaster*: *t* = 2.7, *d*.*f*. = 1, 444, *p* = 0.007). In the other two species, there was no evidence that hemocytes were more likely to be in circulation in resistant populations (Fig 2; *D*. *simulans*: *t* = 0.2, *d*.*f*. = 1, 174, *p* = 0.319; *D*. *mauritiana*: *t* = 1.4, *d*.*f*. = 1,226, *p* = 0.173). Therefore, while an increase in circulating hemocytes underlies resistance in all three species, *D*. *mauritiana* and *D*. *simulans* appear to achieve this by producing more hemocytes, whereas *D*. *melanogaster* appears to mobilise sessile hemocytes into circulation.

To confirm this result we directly counted the number of sessile hemocytes below the cuticle of larvae. We injected the larvae with *E*. *coli* particles that fluoresce when phagocytosed by hemocytes, and 20 minutes later counted the number of hemocytes under the dorsal cuticle (excluding the 8^th^ abdominal segment). In *D*. *melanogaster* we found a significantly fewer sessile hemocytes in the resistant populations (Fig 2; *t =* 5.7, *p* = 4x10^-8^). This contrasts with the increase in circulating hemocytes seen in these populations, and confirms that hemocytes have moved from sessile clusters into circulation. In *D*. *simulans* there was no difference in the number of sessile hemocytes (Fig 2; *t =* 0.1, *p* = 0.89). This confirms that the additional circulating hemocytes in this species result from increased hemocyte production rather than mobilising sessile hemocytes.

### Populations selected for resistance to L. boulardi are more resistant to other species of parasitoid wasps

**Fig 3:**
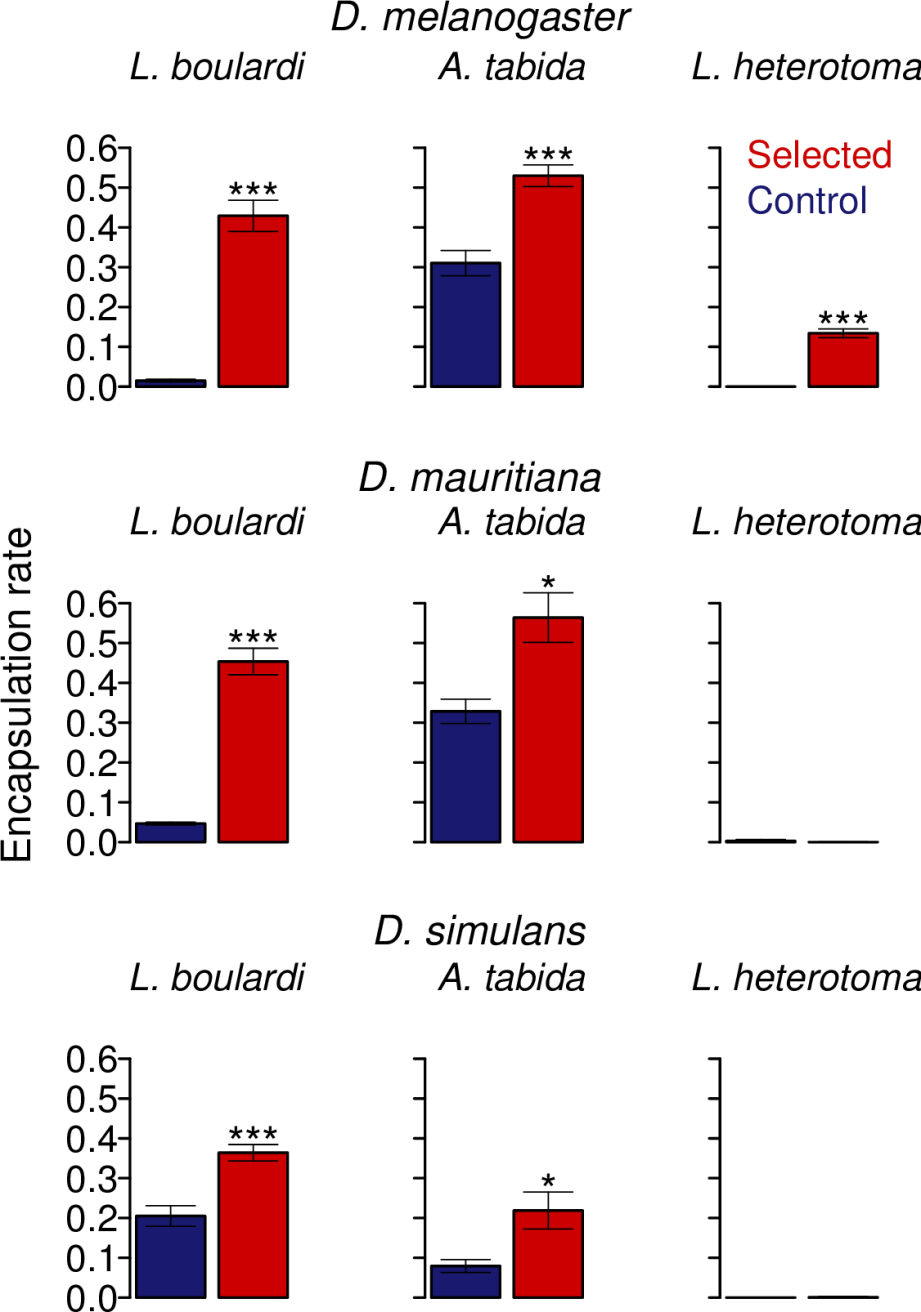
Mean encapsulation rates of selected and control populations challenged with three different parasitoid wasp species. Stars denote the degree of significance of between selected and control populations: * = *p* <0.05 and *** = *p* <0.001. Bars are equal to 1 SE. No. of replicate vials per replicate population = 15 for each parasitoid assayed.

Compared to controls, populations of all three species selected for resistance to the parasitoid *L*. *boulardi* were better able to encapsulate the eggs of the distantly parasitoid wasp *A*. *tabida* (Fig 3; *D*. *melanogaster*: *t* = 5.4, d.f. = 10, *p* = 0.0002; *D*. *mauritiana*: *t* = 3.5, d.f. = 4, *p* = 0.03; *D*. *simulans*: *t* = 2.68, d.f. = 9, *p =* 0.03). This implies a potentially general mechanism for resisting invasion by a parasitoid egg, despite the two parasitoids adopting different strategies to escape the immune response—*A*. *tabida* passively avoids the cellular immune response by attaching to host tissues while *L*. *boulardi* actively sabotages the immune system with venoms and VLPs [53].

In contrast to the assays with *A*. *tabida*, neither control nor selected populations of *D*. *mauritiana* or *D*. *simulans* were able to encapsulate the parasitoid wasp *L*. *heterotoma* (Fig 3; *D*. *mauritiana*: *t* = -1.0, *d*.*f*. = 4, *p* = <0.37). This is notable because *D*. *melanogaster* selected populations encapsulated a significantly larger proportion of *L*. *heterotoma* eggs than control populations (Fig 3; *D*. *melanogaster*: *t* = 13.4, *d*.*f*. = 10, *p* = <0.001), a finding in keeping with previous studies [50]. *L*. *heterotoma* is rarely encapsulated due to venoms that sabotage the cellular immune response [51].

### Evolving resistance is costly in three Drosophila species

**Fig 4:**
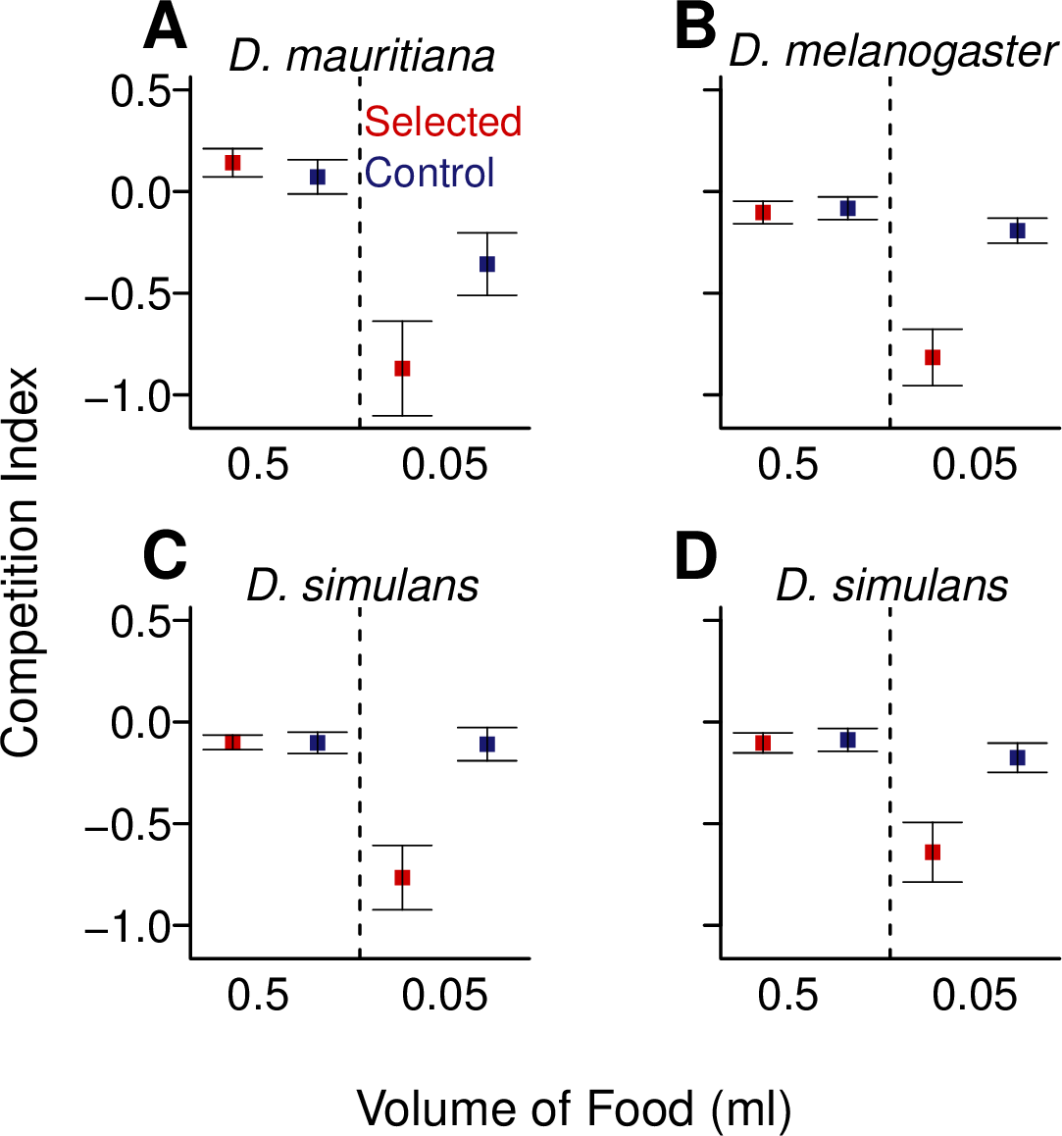
The competitive ability of selected (red) and control (blue) populations in high and low resource environments. The competition index was calculated as a measure of the survival relative to a white-eyed competitor strain (Eq 3). Points represent mean competitive index values of the replicate selected and control populations, calculated from approximately 15 vials. The competitor strain for panels A-C was *D*. *melanogaster* (*w*^1118^) and for panel D was *D*. *simulans* (*w*^501^). Bars are equal to 1 SE.

To examine whether a cost is associated with parasitoid resistance across the three species, we measured the competitive ability of larvae from the control and selected populations. To do this we reared larvae from the population of interest with the same number of larvae from a white-eyed tester strain, and compared their larva-to-adult survival. In a high resource environment with an excess of food there was no difference in the competitive ability of flies from the selected and control populations (Fig4; *D*. *melanogaster*: *F* = 0.5, *d*.*f*. = 1, 10, *p* = <0.504; *D*. *simulans*: *F* = 0.2, *d*.*f*. = 1, 10, *p* = 0.890; *D*. *mauritiana*: *F* = 1.0, *d*.*f*. = 1, 4, *p* = 0.370). To increase the strength of competition we also reared the larvae in a low resource environment with restricted food (1/10^th^ of the food availability of the high resource environment), which resulted in a >70% reduction in survival. In these conditions, the competitive ability of *D*. *melanogaster*, *D*. *mauritiana* and *D*. *simulans* from the selected populations was dramatically reduced relative to the control populations (Fig 4A–4C). Survival of selected populations under low resource conditions was less than half of that for control populations in both *D*. *melanogaster* and *D*. *mauritiana*, and survival of *D*. *simulans* selected populations was 3x less than control populations. This differential ability of selected and control populations to compete in different environments is significant, such that there is an interaction between the resource environment and whether the population was resistant or susceptible for all three species (*D*. *melanogaster*: *F* = 245.3, *d*.*f*. = 3, 20, *p* = <0.001; *D*. *simulans*: *F* = 128.6, *d*.*f*. = 3, 20, *p* = <0.001; *D*. *mauritiana*: *F* = 24.24, *d*.*f*. = 3, 8, *p* = 0.011).

This reduction in competitive ability also does not seem to be linked with a particular tester strain, as in the case of *D*. *simulans* we repeated the experiment using a *D*. *simulans* tester strain (*w*^501^) (Fig 4D). Once again, the selected populations suffered a reduction in survival in the low resource environment (selection regime x environment interaction: *D*. *simulans*: *F* = 163.3, *d*.*f*. = 3, 20, *p* = <0.001) but not the high resource environment (*D*. *simulans*: *F* = 0.4, *d*.*f*. = 1, 10, *p* = 0.542). The result is strikingly similar to the experiments using *D*. *melanogaster* as the tester strain (Fig 4C & 4D).

### Resistance is lost in selected populations under low resource conditions

**Fig 5:**
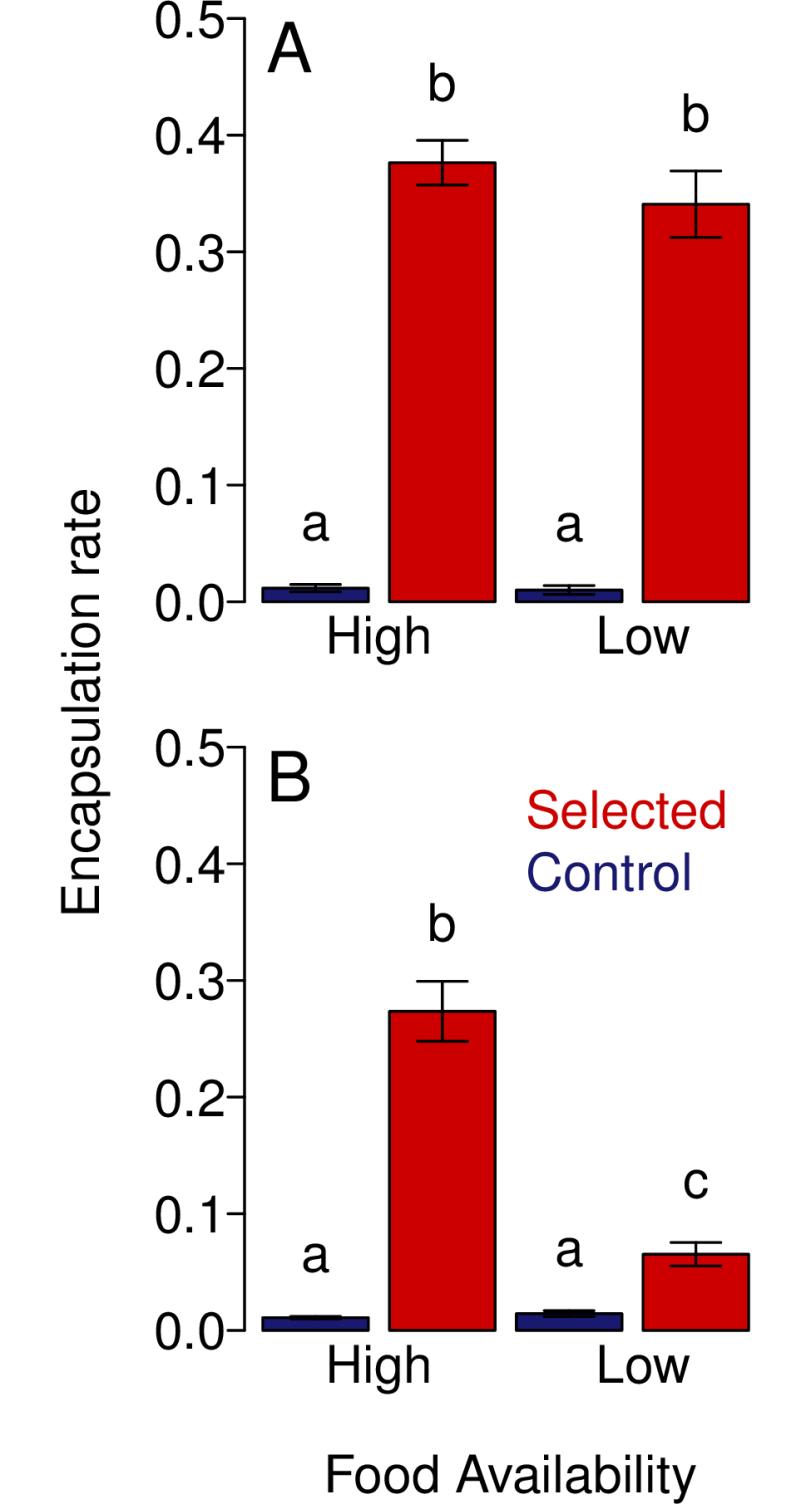
The change in encapsulation rate of populations maintained in high and low food environments. A) The mean encapsulation rates of selected populations split into high and low food treatments at generation 0 prior to maintenance on their respective regimes. B) The mean encapsulations rates of selected and control populations following five generations of maintenance in either a high or low food environment. Bars represent SE. Different letters denotes means that are significantly different from one another, with significance defined as a p value of <0.05 (Tukey multiple comparison test) and these differences are only considering comparisons within a given panel.

In environments where resources are scarce and parasitism rates low, we would predict that selection will favour susceptible genotypes. To test this prediction in *D*. *melanogaster* we split each of our six selected and six control populations in two and maintained one copy of the 12 populations for five generations under ‘feast’ conditions and the other copy of the 12 populations in ‘famine’ conditions. Following five generations, these populations were then expanded for one generation on a standard cornmeal diet and then their encapsulation rates assessed the following generation (Fig 5).

We found that selected populations maintained under a low resource regime displayed a large reduction in their encapsulation rate relative to those maintained under high resources, with encapsulation rate being four times greater in the populations raised on abundant resources (Fig 5B). This interaction between resource availability and whether a population was selected or control had a statistically significant effect on encapsulation rates (Two way ANOVA Selection: Food Regime interaction: *F* = 52.3, *d*.*f*. = 3, 20, *p* = 0.001). This supports the hypothesis that resource availability as well as parasitism rates will determine whether populations evolve resistance.

The encapsulation rates of selected populations raised under a high resource regime declined slightly over 5 generations (Fig 5A left panel versus Fig 5B left panel; Two way ANOVA, Selection: Generation interaction: *F* = 198.4, *d*.*f*. = 3, 20, *p* = 0.017), suggesting that resistance may have had a moderate cost in this environment as well as the low resource environment. Encapsulation rates did not vary significantly between control populations prior to and after the experiment on either food regime, nor was there difference between the selected populations prior to experimentation (Fig 5; Two way ANOVA, Selection: Food Regime interaction: *F* = 21.8, *d*.*f*. = 3, 20, *p* = 0.446).

## Discussion

We found that three species of *Drosophila* could all rapidly evolve increased resistance to the parasitoid wasp *L*. *boulardi*. The gain in resistance and decline in competitive ability was associated with a rise in the number of circulating hemocytes. However, the physiological basis of this differed among species, with *D*. *melanogaster* moving sessile hemocytes into circulation while the other species increased the total number of hemocytes produced.

In all species resistance was extremely costly, with large declines in the survival of flies in competitive resource-poor environments. What’s more, *D*. *melanogaster* selected populations were found to lose resistance more rapidly when maintained on a resource-poor environment.

All three species responded rapidly to selection for resistance. The proportion of individuals encapsulating and killing *L*. *boulardi* eggs doubled after three generations of selection in *D*. *mauritiana* and *D*. *simulans*, and quadrupled in *D*. *melanogaster* (for previous reports in *D*. *melanogaster* see Fellowes et al., 1998 and Kraaijeveld and Godfray, 1997 [3, 27]). Strong selection is likely to be common in nature, as parasitoid wasps can infest over 90% of *Drosophila* larvae [38,54] and successful parasitism always kills flies before they reproduce [33]. Moreover, parasitoid frequency can vary greatly seasonally [38]. As all these species can have generation times of less than two weeks, natural selection could potentially cause dramatic changes in susceptibility over the course of a single season, as has been reported in the crustacean *Daphnia magna* [55].

It is likely that similar immunological changes are causing the increase in resistance in all three species. In all cases, populations that were selected for resistance had over an 80% increase in the number of circulating hemocytes. This is consistent with a previous report that *D*. *melanogaster* populations selected for resistance to *A*. *tabida* have double the concentration of circulating hemocytes [49]. Similarly, resistant *D*. *simulans* lines tend to have more circulating hemocytes [56], and the ability of different *Drosophila* species to resist *A*. *tabida* is correlated with circulating hemocyte number [5,41]. Recent studies investigating *D*. *suzukii*, an invasive species in Europe and the USA, have found an incredible ability to resist native parasitoids and circulating hemocyte counts around ten times that of *D*. *melanogaster* [57–59]. Together these results suggest that there are strong evolutionary constraints, such that *Drosophila* must increase circulating hemocyte numbers in order to evolve parasitoid resistance.

The tight association between hemocyte number and resistance is not unexpected. Hemocytes are specialised immune cells that detect, bind to, encapsulate and ultimately kill parasitoid eggs [60–62]. Injection of a parasitoid egg into a *Drosophila* larva usually results in an increase in circulating hemocyte numbers [41,56,63,64], so it is possible that the cellular immune system of the resistant populations is in a constitutively activated state. The anti-parasitoid response involves the differentiation of a specialised hemocyte type known as lamellocytes from prohemocytes in the lymph gland and plasmatocytes in the sessile clusters [44]. Before lamellocyte differentiation, circulating plasmatocytes adhere and spread around the parasitoid egg, forming the first capsule layer [65]. Thus, having a large pool of circulating plasmatocytes to draw on following parasitoid invasion likely aids the encapsulation process [41].

Despite all three species evolving resistance by increasing circulating hemocyte numbers, the developmental basis of this change is not the same. In *Drosophila* a large proportion of hemocytes are in sessile clusters. When *D*. *mauritiana* and *D*. *simulans* evolved resistance, they increased total hemocyte numbers, resulting in more hemocytes in present circulation as well as in sessile clusters. This is analogous to employing more soldiers in the military. In contrast, when *D*. *melanogaster* evolved resistance the number of circulating hemocytes increased, but the total hemocytes remained the same. This is equivalent to having more of your soldiers out on patrol and less in the barracks. Therefore, we have shown that parasitoid resistance is a case of parallel evolution, where three independent species evolve similar traits in response to the same selection pressure. At the phenotypic level the similarities are striking. In all cases, the parasitoid is being killed by encapsulation, resistance comes at a cost to larval competitive ability, and the number of circulating hemocytes increases. However, the developmental origins of this difference are different in *D*. *melanogaster* than the other two species. A key unanswered question is whether parallelisms extends to the genetic level—are similar pathways, genes or even genetic variants responsible for resistance in the three species?

Parasitoid resistance appears to be a universally costly trait. When reared in a low-resource high-competition environment, the survival of resistant flies in all three species was reduced by over 70% relative to controls. This is likely driven by low survival of resistant flies when protein or other nutrients are in short supply. Similar costs have previously been reported in *D*. *melanogaster* populations selected for resistance to *L*. *boulardi* [27] and *A*. *tabida* [3], where resistant larvae have been found to have lower feeding rates [12]. Our results demonstrate that this is not a quirk of this species. Instead, there is a strong underlying evolutionary constraint that results in resistance being costly across multiple species. This cost to resistance likely explains why genetic variation in susceptibility is maintained in nature, as any resistance allele that was cost-free would likely have a strong advantage and be fixed by selection.

Evolving resistance to parasites may be costly if resources are diverted from other functions into immune defences. When Kraaijeveld et al first observed increased hemocyte numbers in parasitoid-resistant *D*. *melanogaster* larvae, they argued that the resource cost of increasing hemocyte production likely explained the cost of resistance [49]. As resistant larvae feed at a slower rate, they speculated that this resource competition could be the result of hemocytes and the head musculature being derived from the same embryonic tissue [49]. However, we have found that in resistant populations of *D*. *melanogaster* there is no increase in the number of hemocytes produced, so this cannot be the reason resistance is costly. Instead, the increased number of circulating hemocytes may reflect a constitutively activated cellular immune system. If this is the case, then the costs of resistance could result from autoimmune damage.

In all three species the cost associated with parasitoid resistance is far greater in low-resource conditions where larval competition is intense. Therefore, population or species level differences in parasitoid resistance might be driven by differences in resource availability (or other stresses that affect the cost of resistance) as well as differences in parasite pressure. This is supported by our finding that resistance was rapidly lost when populations were maintained under resource-poor conditions, with a ~75% drop in encapsulation rates after just five generations in this environment. Thus whether resistance is favoured in a given *Drosophila* population will be tightly linked with the locally available resources. This finding might also explain some why some *D*. *melanogaster* populations in unusual environments like sherry cask slime and indoor fruit markets are anomalously resistant [33,40]. It is likely that these costs will be expressed in nature, as it is thought that resources are far more plentiful in laboratory conditions than in natural populations of *Drosophila melanogaster*. In adult females, levels of virtellogenesis and hence fecundity are far lower in the wild than in the lab, reflecting restricted access to food [66]. Larvae seem to similarly suffer from nutrient limitation, as flies reared in the lab are larger and have more ovarioles than flies in nature [66].

Natural *Drosophila* populations encounter multiple parasitoids. We found that all three species selected to resist *L*. *boulardi* were also more resistant to *A*. *tabida*. Given that *D*. *melanogaster* populations selected for resistance to *A*. *tabida* have an increased number of circulating hemocytes [49], it is likely that increased hemocyte numbers are the cause of the correlated increase in resistance to *A*. *tabida* in our experiments. Patterns of cross resistance have previously been explored in more detail in *D*. *melanogaster*. In line with our results, *D*. *melanogaster* populations selected for resistance to *L*. *boulardi* have previously been shown to be resistant to *A*. *tabida* and *L*. *heterotoma* [50]. However, the reverse is not true and *A*. *tabida* selected lines are not significantly more able to encapsulate *L*. *boulardi* than control populations [50], although they are better able to encapsulate *L*. *heterotoma*. This suggests a specific and a general component to parasitoid resistance, with *L*. *boulardi* resistance requiring both factors and *A*. *tabida resistance* only requiring the general component [5,50,67]. These patterns of cross resistance will have important consequences for the evolution of natural populations, with the community of parasitoids present determining levels of resistance.

Curiously, selection for *L*. *boulardi* resistance led to a correlated increase in *L*. *heterotoma* resistance in *D*. *melanogaster* but not *D*. *mauritiana* or *D*. *simulans*. Neither selected nor control populations of these species ever survived parasitism by *L*. *heterotoma*. All three species use lamellocytes as the main anti-parasitoid immune cell and *L*. *heterotoma* virulence factors destroy lamellocytes that are present in the hemolymph and also appear to damage the machinery of hemocyte production [64,68,69]. *L*. *boulardi* virulence factors, in contrast, appear to block hemocyte release and morphologically alter lamellocytes making them non-functional [68]. These differences in action could explain the lack of cross resistance if *D*. *mauritiana* and *D*. *simulans* are especially susceptible to these venoms, although this then poses questions regarding how *D*. *melanogaster* selected populations are able to overcome this.

We have sampled each species from a single geographical location, and it is known that there is strong geographical variation in parasitoid resistance in *Drosophila* [33]. It is therefore possible that some of the differences that we observe between species may also exist between populations of the same species. Similarly, some of the patterns that are the same across species may not hold when new populations are sampled. These questions await future study.

From this work we draw three main conclusions. (1) Costs of resistance maintain genetic variation in susceptibility to infection. These costs can explain why all three species of *Drosophila* remain susceptible in nature despite it being easy to select for resistance in the lab. As these costs are found across all three species, it suggests that there are fundamental constraints to evolving resistance. (2) Whether resistance evolves will depend not only on parasitism rates but also food-availability and the community composition of parasites. (3) Different species all evolve resistance by increasing investment in cellular immune defences, although the convergent evolution of the immune phenotype has different developmental bases in different species.

## Materials and methods

### Founding of outcrossed populations prior to selection

A *D*. *melanogaster* outcrossed population (COP2) was founded in 2014 from 2050 isofemale lines. These lines were founded by flies collected from 10 separate field sites around Coventry, England (52.383807°N, -1.481671°W; 52.386305°N, -1.484438°W; 52.386827°N, -1.480226°W; 52.412142°N, -1.466066°W; 52.41714°N, -1.601703°W; 52.386701°N, -1.481095°W; 52.386921°N, -1.482000°W; 52.410799°N, -1.468799°W; 52.386345°N, -1.483517°W; 52.408893°N, -1.582120°W). Females were sorted and placed into vials containing *Drosophila* food [70] in order to establish isofemale lines. The progeny of these isofemales lines were then collected and five flies from each line pooled to found an initial outcrossed population of ~10,250 flies. The progeny of this initial generation were immediately used for selection.

Similarly, *D*. *mauritania* Outcrossed Population (MOP) and the *D*. *simulans* Outcrossed Population (SOP) were founded using a similar technique, with the caveat that previously established isofemale lines that had been maintained in the laboratory were used, rather than wild caught lines. 36 *D*. *mauritiana* (provided by Marie-Louise Cariou, Mauritius, [71]) and 180 *D*. *simulans* (collected in North America and provided by Trudy Mackay) isofemale lines were combined to create MOP and SOP, respectively. Both populations were put through an intermediate step of producing sub-populations in an attempt to maintain genetic variation. Sub-populations were created by pooling five lines together into a population for a single generation. The progeny of these sub-populations were then aggregated to create a large outcrossed population. Both MOP and SOP were maintained as large outcrossed populations for approximately 20 and 8 generations prior to selection, respectively.

### Drosophila and parasitoid wasp stock maintenance

Unless otherwise stated, all *Drosophila* were maintained on a cornmeal diet [70], supplemented with a sprinkling of dried live yeast, 70% relative humidity and a 12hr:12hr light-dark cycle. Parasitoid wasps *L*. *boulardi* strain from Sienna, Italy (NSRef [72]), *L*. *heterotoma* (collected in Oeiras, Portugal in 2014) and *A*. *tabida* (collected in Sainte Foy-Lès-Lyon, Rhône, France in 2012 and provided by Fabrice Vavre) were maintained on an outcrossed *D*. *melanogaster* population, and cultivated at 25°C. A single wasp was placed on eggs collected from COP1 and left for 72 hours before removal. Following emergence female adult parasitoids were stored on apple agar vials (apple juice concentrate, agar, glucose, water, nipagin) with males at a ratio of roughly 2:1 at 18°C, with humidity maintained at 70% and a 12hr: 12hr light and dark cycle.

### Assessing the frequency of encapsulation within a population

Adult flies were placed in a population cage and provided with apple juice agar plates (apple juice concentrate (120ml), agar (8g), glucose (0.4g), water (440ml), nipagin 10% w/v (10 ml)) with fresh yeast paste applied to the surface. They were left for five days prior to experimentation to ensure sufficient time for remating. Following this period, the plates were changed and flies were allowed to lay over a period of twenty four hours. These plates were then collected and the eggs removed through surface washing with PBS and gentle stimulation with a soft bristle paint brush. Eggs were then transferred into a 50ml falcon tube and the suspension left to settle for 1 minute. 1 ml of dense egg suspension was then transferred from this tube into a clean 1.5ml microcentrifuge tube. To set up experimental vials, 5μl of dense egg suspension was then added to each cornmeal vial, which were subsequently numbered and randomly assigned to a treatment.

A single female wasp of age 3–5 days for the parasitoids *L*. *boulardi* and *L*. *heterotoma*, or 7–9 days for *A*. *tabida*, was added to each parasitised treatment vial. This was done 10 hours after the eggs were added to the vial, so the fly larvae were aged between 10-34hrs. Control vials were left unparasitised. All vials placed in a 25°C controlled temperature (CT) room, with humidity maintained at 70%. Wasps were removed after 24hrs for *L*. *boulardi* and *L*. *heterotoma*, or 48hrs for *A*. *tabida*. Vials were left to develop for a total of 14 days. Following this, flies were sorted and counted under CO_2_ anaesthesia, and then flies from parasitised vials were crushed between two clear microscope slides and checked for the presence of capsules and the number of flies with at least one capsule present recorded. As a precautionary measure every tenth control vial was also checked in this way, although no capsules were ever discovered in these vials.

### Estimating the encapsulation rate

The Parasitism rate (*Pr*), which we define as the proportion of *Drosophila* larvae infected with at least one parasitoid egg, was estimated by comparing the mean number of adult flies in parasitised (*Nt*) and unparasitised vials (*Nc*) [72]. The mean number of flies containing at least one capsule (*Ncap*) was used to account for flies that had survived parasitism.

$$Pr=\frac{Nc-\left(Nt-Ncap\right)}{Nc}$$ (1)

The Successful Encapsulation Rate (*SER*), defined as the proportion of parasitised flies surviving into adulthood [72], was calculated as:

$$SER=\frac{Ncap}{Pr\times Nc}$$ (2)

### Selection for resistance

We artificially selected for resistance by exposing the populations to parasitoids over 6 generations and only allowing flies that displayed visible evidence of having survived parasitism to reproduce. Using the method described above, 5μl of eggs from the outcrossed populations were placed into vials of food (see above), which were assigned randomly to be parasitised and as controls. A single female *L*. *boulardi* wasp was placed into each vial to be parasitised and removed after 36 hours. This long period of parasitism was decided upon for reasons of experiment feasibility. All vials were matured at 25°C for 14 days. Emerging flies were sorted on CO_2_ and parasitised flies were sorted under a dissecting microscope (Leica MZ6) at 20x magnification and those identified as possessing a capsule isolated. Presence of a capsule is taken as affirmation of both parasitism and a successful immune response.

To establish populations for selection, capsule containing flies were randomly sorted into six populations. Control populations were established in the same way from the unparasitised vials. Further selection was carried out in the same way for each population each generation, with selected populations being parasitized and only flies containing a capsule allowed to continue. At each generation the population size was kept constant for all populations, and this was always above 180 individuals per population with an approximate sex ratio of 50:50. Selection was carried out continuously for six generations, with the encapsulation rate of the selected populations assayed each generation. Control populations were assayed at generations one and six. Following generation six, selection was relaxed and the populations maintained with large population sizes, with selection carried out only every three generations for reasons of convenience. Selected and control populations were maintained at a population size of 1500 per line in cornmeal media vials [70] at a density of ~50 larva per vial in a 25°C controlled temperature (CT) room, with relative humidity maintained at 70%.

### Assessing the specificity of parasitoid resistance

We investigated whether populations selected for resistance to *L*. *boulardi* exhibited increased resistance to *L*. *heterotoma* and *A*. *tabida*. Vials containing 5μlof eggs from each population were set up as described above and assigned to one of four treatments: controls and parasitised by *L*. *boulardi*, *L*. *heterotoma*, or *A*. *tabida*. Larva of ages 24–48 were then parasitised by a single female wasp. Wasps were removed after 24 hours for the *Leptopilina* species and 48 hours for *A*. *tabida*, and then left to develop at 25°C 70% relative humidity for 14 days. The adult flies were then sorted on CO_2_, checked for the presence of capsules, and the encapsulation rate estimated using Eq 2.

### Counting hemocytes

To count hemocyte numbers, *Drosophila* larvae were reared by allowing selected and control populations to lay on a 90mm apple juice agar plate with live yeast paste added to the surface for four hours, and the resulting eggs collected into an Eppendorf tube as above. Following this, 8μl of egg mixture was transferred to 55mm plates containing standard *Drosophila* cornmeal food, the surface of which had been scored with a needle. The plate was then left for 72 hours, so the larvae were between 72–76 hours old. Larvae were then removed from the plates using forceps, washed in PBS and then dried on filter paper to remove any food contamination. For an estimate of larval ‘Circulating hemocyte number’ a single larvae was gently bled by tearing the ventral cuticle using a pair of fine steel forceps while immersed in 4μl of Ringer’s solution (NaCl 46mM, KCl 182mM, CaCl2 3mM, TrisBase 10mM, pH = 7.2) on top of a grid (10-by-10 of 0.4mm^2^ squares). The number of cells was counted under a compound microscope (Leica DM750). For ‘Total hemocytes number’ larvae were first rolled ~20 times with a brush to physically dislodge hemocytes residing in sessile clusters into circulation [52]. This method of dislodging haemocytes from the sessile clusters, similar to that described in Petraki *et al*., (2015), recovers approximately 60% of total haemocytes (Figure A in S1 Supporting Information), over four times as many haemocytes as are found in circulation without disruption. The hemocytes were collected as described above.

To visualize sessile hemocytes in the dorsal cuticle, 69nl of pHrodo Red *E*.*coli* BioParticles (Life Technologies) were injected into the larval haemocoel using a nanoinjector (Nanoject II, Drummond Scientific). Injected larvae were incubated for 20min in fly food, washed in PBS, dried in filter paper and immobilized between two glass slides. Pictures were taken with fluorescent stereomicroscope (Leica MZ16F) equipped with a monochrome digital camera (Leica DFC340 FX). Hemocytes in the dorsal cuticle were counted manually with ImageJ. Hemocytes in the A8 segment were excluded because the high hemocyte density in this region makes it very hard to distinguish cells. To avoid biases with manual counting, files names were renamed with random tags blindly to the experimenter counting the cells.

### The competitive ability of selected populations

To examine whether selection for resistance produced a correlated decline in larval competitive ability, we measured the survival of larvae reared in competition with a standard tester strain. All three *Drosophila* species were competed against an isogenic white-eyed *Drosophila melanogaster* line (*w*^1118^), and *Drosophila simulans* was additionally tested with an inbred white-eyed *Drosophila simulans* line *w*^501^ [73]. Larval competitive ability was assessed using two different quantities of food. Yeast paste was prepared by adding a mixture of 25g Allinson’s dried live baker’s yeast per 100ml water, with 0.5ml (approximately 0.06g protein) and 0.05ml (approximately 0.006g protein) of yeast paste added to 50mm apple juice agar plates for ‘high’ and ‘low’ food types respectively. Plates were covered and left to dry overnight at room temperature. Previous studies have shown that these two volumes represent both a highly stressful (low food) and non-stressful (high food) environment for flies [3,27]. To assess competitive ability, fifteen second instar larvae of both the experimental and the tester strain were added to plates from the high and low food treatment, and left to develop at 25°C for 12 days. There were 20 replicate vials per treatment for each population. Flies were then sorted on CO_2_ and the number of white eyed tester flies and red eyed experimental flies that survived to adulthood recorded.

### Food availability and the maintenance of parasitoid resistance in *D*. *melanogaster* populations

To investigate how resistance might be maintained in natural populations, we generated replicates of our *D*. *melanogaster* selected and control populations and allowed these replicate populations to propagate for five generations without selection under different environmental conditions. Each population was maintained on two different food resource regimes: a rich or poor food resource. In order to produce these conditions, either 1ml or 50μl of 25% yeast was pipetted onto the surface of 50mm apple agar plates, representing the rich and poor food sources respectively.

Each generation flies were collected and placed in a population cage and allowed to outcross for 24 hours. Following this, flies were allowed to lay onto a 90mm apple agar plate with 1ml of 25% yeast paste over a period of 12 hours. These eggs were then collected in a 1.5ml Eppendorf tube and suspended in PBS. 5μl of these eggs were then transferred to each treatment plate. Population size for each population in each treatment was maintained above 100 individuals each generation. Populations were maintained at 25°C for 18 days. The longer period of time was required due to the effect of low resources on development time.

Following five generations, both high and low resource populations were then expanded for one generation on a standard cornmeal media with a sprinkling of dry yeast, and in the subsequent generation their encapsulation rate estimated using the methods described above and Eq 2. Populations’ encapsulation rates were also estimated at generation 0 prior to rearing on the two different food regimes.

### Statistical analysis and data availability

All statistical analyses were performed in using R. The raw data and scripts to perform the analysis and plot the figures are available at the Cambridge data repository https://doi.org/10.17863/CAM.13612. The encapsulation rates for selected and control populations of *Drosophila* were compared by first calculating the encapsulation rate *SER* for each population (Eq 2), and comparing these estimates between the two types of population using Student’s *t*-test.

Differences in the number of hemocytes in selected and control larvae were compared using a generalised linear model (glm). We assumed a Poisson distribution, under quasi-likelihood estimates to account for over-dispersion of the data. The final model applied to the data was Hemocyte Count ~ Partition * SS. Where Partition represents whether the hemocyte count originated from larvae who had undergone cluster disruption or not, and SS, the selected state.

To compare the competitive ability of selected and control flies, we followed Kraaijeveld and Godfray [3] by first calculating a competition index (*CI*) for each plate of flies [74]:

CI=ln(e/t+1) (3)

where *e* is the number of adult experimental flies and *t* is the number of adult white-eyed tester flies. Differences between mean competitive indexes of the resistant and control populations were assessed using Student’s *t* test.

## Supporting information

S1 Supporting Information Supplementary methods and results. (PDF)
