# Supplementary material for: Parallel and costly changes to cellular immunity underlie the evolution of parasitoid resistance in three Drosophila species
Source: PLoS Pathog. 2017 Oct 19;13(10):e1006683. doi: 10.1371/journal.ppat.1006683 (PMC5663624; doi:10.1371/journal.ppat.1006683)
Supplement: S1 Supporting Information — (PDF) [file ppat.1006683.s001.pdf]

Supporting Information

	Encapsulation rate				
	Source population	Generation one		Generation six	
		Selected	*Control*	*Selected*	*Control*
D. melanogaster	0.011	0.032	0.036	0.353	0.028
D. simulans	0.126	0.126	0.107	0.371	0.100
D. mauritiana	0.096	0.129	0.093	0.384	0.075

Table A. Encapsulation of parasitized flies that successfully encapsulated the parasitoid wasp *L. boulardi*. Estimates were calculated for the outcrossed starting population for each species and for the selected and control populations at generation one and six. The generation one and six data is also shown in Figure 1 if the main text.

Cluster disruption protocol efficiency:

To determine the efficiency of the cluster disruption protocol, we have measured the number of circulating hemocytes, sessile hemocytes and circulating hemocytes after cluster disruption in an isogenic stock that expresses the fluorescent nuclear marker DsRed in all hemocytes (Hml-DsRed) (Clark et al 2011). This was achieved by modifying the protocol described in (Petraki et al 2015). To estimate the number of circulating hemocytes, 72 hours old larvae were bled by cutting the ventral cuticle in a Pap-pen well with ~10µl of of PBS. Hemocytes were incubated for 30min in a humid chamber, a cover slide was placed on top of the slide and one picture was taken on a Leica DM6000B florescence microscope. Hemocyte nuclei were counted with ImageJ (Schindelin et al 2012). To estimate the number of hemocytes in circulation after cluster disruption, the same protocol was used with larvae that were previously rolled with a paintbrush ~20 times, as described above. To estimate the number of sessile hemocytes in the cuticle, larvae were firstly bled to remove circulating hemocytes. In a Pap-pen well with ~10µl of PBS, hemocytes from the cuticle were scraped and allowed to settle for 30min before a picture was taken. In a second well, the larval cuticle was spread on the slide and a second picture was taken to estimate the number of remaining hemocytes.

Differences between treatments were assessed using log(hemocyte number) and modelled under a one-way ANOVA. Model = log(Cell Number) ~ Treatment.

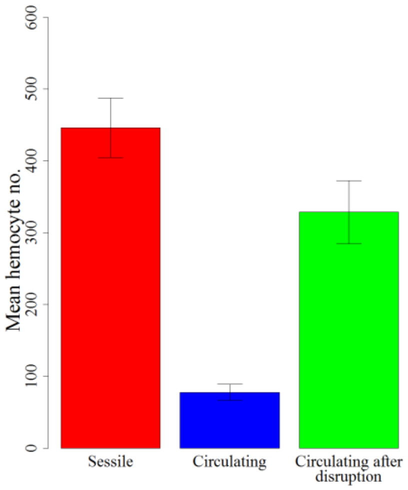

Figure A. Mean hemocyte number of single larvae estimates using different methods of quantification. Sessile represents the number of hemocytes found in sessile clusters, circulating hemocytes represents the number of hemocytes found in circulation following bleeding, and circulating after disruption represents the number of hemocytes found in circulation after a larvae has been rolled 20x with a paintbrush – dispruting sessile hemocytes into circulation. Bars represent SE. N = 24 for each treatment. The methods to generate this data are above.

Supplementary references:

Schindelin J., Arganda-Carreras I., Frise E., Kaynig V., Longair M., Pietzsch T., Preibisch S., Rueden C., Saalfeld S., Schmid B., Tinevez J. Y., White D. J., Hartenstein V., Eliceiri K., Tomancak P., Cardona A., 2012. Fiji: an open-source platform for biological-image analysis. Nat. Methods, 9, 676–682, doi: 10.1038/nmeth.2019.

Clark R. I., Woodcock K. J., Geissmann F., Trouillet C., Dionne M. S., 2011 Multiple TGF-b Superfamily Signals Modulate the Adult Drosophila Immune Response, Curr. Biol., 21, 1672–7, doi:10.1016/j.cub.2011.08.048

Petraki, S., Alexander, B., Brückner, K, 2015. Assaying Blood Cell Populations of the Drosophila melanogaster Larva. J. Vis. Exp. 105, e52733, doi:10.3791/52733
